# IL28B gene polymorphism SNP rs8099917 allele GG is associated with HTLV-1-associated myelopathy/tropical spastic paraparesis (HAM/TSP) in HTLV-1 carriers

**DOI:** 10.1186/1742-4690-11-S1-O18

**Published:** 2014-01-07

**Authors:** Tatiane Assone dos Santos, Fernando Vieira de Souza, Karen Oliveira Gaester, Daniela Cardeal da Silva, Luiz Augusto Marcondes Fonseca, Olinda do Carmo Luiz, João Renato Rebello Pinho, Alberto JS Duarte, Augusto Penalva de Oliveira, Jorge Casseb

**Affiliations:** 1 Laboratory of Dermatology and Immunedeficiencies, Department of Dermatology, University of São Paulo Medical School, Brazil; 2 Institute of Tropical Medicine of São Paulo, São Paulo, SP, Brazil; 3 Department of Preventive Medicine, University of São Paulo Medical School, Brazil; 4 Institute of Infectious Diseases “Emilio Ribas” (IIER) of São Paulo, São Paulo, SP, Brazil

## 

The polymorphism of IL28B was described as important in the pathogenesis of infections caused by some viruses. The aim of this research was to evaluate whether IL28B gene polymorphisms (SNP rs8099917 and SNP rs12979860) were associated with HAM/TSP. The study included 144 subjects were classified according to their neurological status in two groups: Group I (60 asymptomatic HTLV-1 carriers) and Group II (84 HAM/TSP patients). Blood samples were collected, and PBMC separated by Ficoll density gradient centrifugation. DNA was extracted using a commercial kit, and the DNA was stored at -80°C for later analysis. After this, the proviral load was quantified, and the rs8099917 and rs12979860 SNPs in the region of IL28B-gene were analyzed by StepOnePlus Real-time PCR System. A multivariate model analysis, including gender, age, and HTLV-1 DNA proviral load, showed that IL28B polymorphism SNP rs12979860 was not independently associated with HAM/TSP outcome. In contrast, the SNP rs8099917 allele GG was independently associated with HAM/TSP outcome (OR=6.25; IC95%=1.22– 32.00). Persons with SNP rs8099917 genotype GG may present a distinct immune response against HTLV-1 infection. So, it seems reasonable to suggest that a search for IL28B polymorphisms should be performed for all HTLV-1-infected subjects in order to monitor their risk for disease development; however, since this is the first description of this finding in the literature, we should first replicate this study with more HTLV-1-infected persons to strengthen the evidence already provided by our results.

